# Address

**Published:** 1876-03

**Authors:** W. W. H. Thackston


### ARTICLE II.

### *Address.*

BY W. W. H. THACKSTON, M. D., D. D. S.

Delivered before the Virginia State Dental Association.

*Gentlemen of the Virginia Association of Dental Surgeons:*

Again it is our duty and privilege to utter our anniversary greetings and salutations; to bid you once more welcome to these Halls, and to the rich banquet in which you ll will participate, and to which you all may contribute.

We open our heart and stretch out our arms, that we may gather you in and enfold you all in the bonds of professional unity and brotherhood, with the hope and trust that, forgetting or laying aside all professional jealousies and estrangements, all the petty grievances and annoyances that disturb the harmony and ripple the currents of professional life, we will here meet on consecrated ground, an around our altars brighten the links and strengthen the cords of fraternal union and brotherly sympathy.

We do not mean that we expect you all to think alike upon every subject that may engage our attention as dental surgeons, to hold the same convictions, to cherish the same doctrines, or adopt the same modes of practice in all given cases; that would be unreasonable and undesirable. Unreasonable, because men are so constituted as to see the same object in different lights, to receive varying impressions from the same causes, and to adopt diverse and sometimes opposite methods of reaching the same result. Undesirable, because all interest would be abated, and all progress retarded or arrested, were thought and action confined to grooves and circles, and one monotonous treadmill path bind and circumscribe our professional efforts and activities.

In a certain sense we expect, nay more, we desire that you should differ and disagree, that you may the more earnestly and thoroughly discuss and investigate unsettled questions, more carefully compare observations, authorities and opinions, and more closely and satisfactorily solve the problems that claim our consideration as members of an important and advancing profession.

Out of such differences and disagreements will be evolved the power and stimulus to additional progress and higher improvement; our energies will be aroused and our steps quickened in that advance, which is now the watch-word and rallying cry in every department of science.

But above and around all that is variant in sentiment and opinion, as to theories or points of practice, as to alleged improvements or important discoveries, we would hang the mantle of brotherly association and personal friendship. However our interests may antagonize and conflict in the broad arena of professional life; here at least, they are identical and in full accord; and if we have grievances that we remember, or resentments that we have cherished, let us generously and magnanimously lay them all upon our common altar, for our individual and for the general good; and striking hands and standing shoulder to shoulder, let us move in concert and in harmony to the great work of advancing the division of science we assume to represent; and which challenges our most earnest efforts for its culture and development.

There appears, gentlemen, at present, a most singular misapprehension, and in some quarters, a most painful uncertainty as to the status and relations of dental surgery as a profession; and in the minds of some of the contributors and managers of our journalism, most unsettled and conflicting sentiments and convictions in regard to our educational resources and facilities If we are to receive some of the utterances of our *Journals* as indices of public opinion, it would seem that it is yet an open question whether dental surgery is a business, a trade, an art or a profession, and whether after the splendid results of a forty years experiment, we should not now abandon our distinctive educational system—suppress our schools and colleges and attach ourselves to the purely medical institutions of the country. Whether we should not surrender our college degree as a "badge of partial culture," and look to the medical schools as the source of instruction—the fountains of honor and dignity, and to the M. D. as the "badge" of thorough and complete culture and qualification for our offices and duties, and as "dentists of the period."

We have thought, that perhaps we could not better improve the present hour and occasion, than by submitting a few observations and reflections upon these two propositions. We wish you, gentlemen, to know where you stand, what plane and position you occupy, and by what tenure you hold social and public recognition as members of a liberal profession. We wish also, to avert a calamity which may threaten, if it does not already impend, for we really and sincerely believe, that any radical change in our present educational system, would be fraught with the most deplorable consequences, both to ourselves as dentists and to society at large.

But first, are we a professional body? or do we simply comprise an association of artizans, mechanics and tradesmen? The resolution of the "American Medical Association," declining to admit as members of that body dentists *as dentists*, together with the flippant and vulgar criticism of one or two medical journals, stigmatizing our college degree as a badge of "partial culture," has not only disturbed the equanimity, but shaken the judgment of some of our ambitious and aspiring brethren, and they appear to stand ready to make any concession, to submit to any "snubbing," and to embrace any expedient, however disastrous and humiliating, that may secure recognition and bring them into acknowledged affilliation with the medical profession and its organized societies.

Now, gentlemen, in the outset we have to observe that we yield to none in a proper and becoming respect and admiration for medicine as a science—as an elevated and honorable profession, and as a necessary and benevolent pursuit. We cherish the highest veneration for the good and the great men who have illustrated, and who are now illustrating the annals of medicine by their skill, their talents, their learning and their benefactions; and we regard with indescribable interest and satisfaction, the research, the advances, and the improvements that distinguish the present era in medical progress and history. But while cheerfully admitting and according all that can be justly and fairly claimed for the profession of medicine, we submit and maintain that dental surgery, a closely allied but distinct science and pursuit, has now all the elements and resources of an independent, self-sustaining and progressive liberal profession.

We have an educational system which we shall presently attempt to discuss; we have standard text-books; incorporated and successfully conducted schools and colleges, and a journalism and literature, respectable, creditable and available for all our present wants and needs. We are yearly recruiting and reinforcing our ranks with a better and more cultivated class of men, and many of our veterans are gathering laurels and achieving distinction and world-wide reputations from their investigations, researches and improvements; and however much it might tickle the fancy of some of our brethren, to be received and acknowledged as members of the "American" or some other medical association, and however much it might pander to the vanity and egotism of the small men amongst our medical confreres, to play condescending patron to the dentist; we confess ourselves unable to perceive that either dental surgery, or medicine, as sciences and professions would be particularly or specially benefited by the alliance or combination.

We submit, that dental surgery can now afford to stand upon its present record, to assert its own claims to consideration, and to challenge the confidence and respect of society, and of all other professions, medicine included; and we further submit, that this is the only position that will command the respect and consideration which an elevated profession can confer, and which an elevated profession can afford to accept.

But we are told that we are not recognized as qualified delegates and members of the medical societies, and our college degree, our "D. D. S," is regarded as a badge of "partial culture," a symbol of individual and professional inferiority and subordination." What is to be done! exclaim some of our dissatisfied and ambitious brethren; "had'nt we better change our educational system, and abolish our own colleges? had'nt we better drop the college degree and hide it away from public view, and all become M. D. dentists? thus bridging the fearful chasm between dental surgery and medicine." Now let me ask of what consequence or concern to us, is the action of the

American Medical Association? For reasons satisfactory to that body, it has declined to receive dentists, and the representatives of our colleges as members—for similar or for other reasons, we may refuse physicians and the representatives of medical schools, membership in our conventions and associations; and we could do so upon established precedents, without a violation of the amenities and proprieties that distinguish professional intercourse. We do not regard it as a strained analogy when we direct attention to our various church organizations; no Protestant has less respect for himself and his own church, because the Catholic will not permit him to sit in his councils, and no Episcopalian feels himself degraded or dishonored, because his Jewish neighbor refuses him a high seat in the synagogue or the sanhedrim; and why should a dentist of common sense or ordinary intelligence feel himself humiliated and his profession abased, because the doors of the medical societies are not thrown wide open to all who may wear the title of "doctor." We apprehend that the doctors of divinity, of law, and of philosophy, would be regarded quite as ineligible to membership in the medical associations as are the dentists, but we should confess to an amazement we have no language to express, were they to place themselves in the unenviable and ridiculous attitude assumed by some of our self constituted leaders.

But some coxcomb of an editor, some conceited puppyish journalist, has disparaged our college degree, has sneered at it "as a badge of partial culture," and straightway some of our dentists and journalists, go into hysterical spasms—and as a "sop to Cerberus," as a propitiatory offering to the gods of medicine, (for there are a great many of them now-a-days, and *æsculapius* is nowhere,) propose to recast and remodel the whole machinery of dental education, and dental practice and usage, to separate operative and artificial dentistry as incompatible, and to divorce from both what is styled "par excellence," and just the thing: "Oral Surgery" and "Oral Medicine."

True professional progress demands no abandonment of principle, no sacrifice of manly self-respect; and if ever recognized at all we apprehend the "oral surgeons" and "oral physicians" would be given back seats in the medical associations, would soon be glad to drop their new born title, and rejoice to be again acknowledged as respectable dentists.

Our college degree "a badge of partial culture;" partial culture for what? for medicine, for law, for divinity? We admit it our colleges do not propose to educate or confer degrees upon physicians, lawyers or preachers. Partial culture for dental surgery! Then we denounce the assertion and the sneer as false and stupid, and altogether worthy the source in which it had its origin.

Our schools and colleges have only undertaken to cultivate, to teach, and to prepare students for the practice of "dental surgery;" and the degree conferred by those schools can in no just or true sense be called a "badge of partial culture," any more than the M. D., the D. D., or the L. L. D.

For the consolation of our dissatisfied and aspiring dentists, it may be justly said, that each and all of these several degrees, are "badges of only partial culture." The M. D. is taught nothing of dental surgery, and the D. D. nothing of law or medicine, so that these degrees as symbols or badges stand exactly upon the same plane; they are evidences of a given or supposed extent of culture and attainment in each of the several departments of general science, and express and imply no special acquaintance with any department or profession outside the specialties they represent, and must all be regarded as "badges" of a fractional culture in the sum of human learning.

Your degree of "doctor of dental surgery," represents the same value, the same honor, and the same dignity in your profession that M. D. does in medicine, no less and no more, and if you will accept as of any value, the expeence of one who received the third diploma ever issued by a college of dental surgery to a regular graduate, and who has worn this so-called "badge of partial culture," throughout his professional life, it is this,—if you will honor that badge, if you will wear it bright and unstained; it will never degrade or dishonor you or disqualify you as the associate or peer of any man bearing any other badge or symbol, be it M. D., Ph. P., D. D. or L. L. D.

Dental surgery embodies by its legal and social recognition, its schools and colleges, its special literature, its standard text books and journalism, its associations and conventions, all the conditions and requirements of a learned and liberal profession, and as such, has a legal and unquestioned right and privilege, to dispense its distinguishing honors, and confer its special degrees.

Dental surgery ceased to be an art and a trade when the "Baltimore College" was incorporated, and the "American Society of Dental Surgeons" was organized and put in operation; just as medicine and general surgery ceased to be arts and trades, when the celebrated incorporation of "Barber Surgeons of the city of London" was dissolved and was succeeded by the incorporation and establishment of the present "Royal Society of England."

TO BE CONTINUED.

